# Recurrent Urinary tract infections and type 2 diabetes mellitus: a systematic review predominantly in women

**DOI:** 10.3389/fruro.2023.1275334

**Published:** 2023-12-12

**Authors:** Sara B. Papp, Philippe E. Zimmern

**Affiliations:** ^1^ Medical School, University of Texas Southwestern Medical Center, Dallas, TX, United States; ^2^ Department of Urology, University of Texas Southwestern Medical Center, Dallas, TX, United States

**Keywords:** diabetes, recurrent, UTI, urinary infection, type II diabetes

## Abstract

**Background:**

Type 2 diabetes mellitus is considered a risk factor for developing recurrent urinary tract infections. This review examined current knowledge on the incidence rates, bacterial strains, risk factors, treatments, and outcomes of recurrent urinary tract infections in type 2 diabetes, predominantly in women.

**Methods:**

A systematic review was conducted for all English language articles from inception to June 2022 utilizing the Cochrane and Preferred Reporting Items for Systematic Reviews and Meta-Analyses standards in the databases PubMed, OVID Embase, and Cochrane Library. References were cross-examined for further articles. Data collected described the prevalence, characteristics, and management of recurrent urinary tract infections. Risk of bias assessments were performed for all studies.

**Results:**

From 3342 identified articles, 597 met initial study criteria. Fifteen studies from 10 countries were included after full-text reviews. Four studies found higher recurrent urinary tract infection rates in diabetics versus non-diabetics meanwhile others reported recurrence rates from 23.4% to 37%. Four of five studies found diabetes to be a risk factor for recurrent urinary tract infection. *E. coli* was the most frequent causative pathogen. Antibiotic prescription results varied; however, multiple studies determined that longer treatment (≥ 5 days) did not correlate with lower recurrence rates. Risk of bias assessments found the most frequent study weakness to be identification of confounding variables.

**Conclusion:**

This review covered multiple subtopics, with few comprehensive or generalizable results, suggesting a need for more research on how recurrent urinary tract infections can be better evaluated and managed in women with type 2 diabetes.

## Introduction

Urinary tract infection (UTI) is the most common adult bacterial infection in the world, affecting over 60% of women at least once in their lifetime and becoming a recurrent urinary tract infection (rUTI) in more than a quarter of women (1–5). With the growing issue of antibiotic resistance, there is an urgent need to expand our understanding of UTIs, especially recurrent infections (6). Unfortunately, there are few published studies on rUTIs to guide clinical diagnosis and treatment (2, 5, 6).

Patients with type II diabetes mellitus (T2DM) are of special interest to researchers as many studies have shown that individuals with T2DM suffer from UTIs at a higher rate than those without T2DM (7–9). The incidence of diabetes in the US is rapidly increasing, therefore, UTIs are likely to become even more prevalent (10). A 2021 systematic review summarized the current literature on UTIs and diabetes (11). However, there is limited literature that focuses on rUTIs in women with T2DM.

Until recently, there were multiple limitations on rUTI research which contributed to the lack of rUTI studies in various populations. Criteria for rUTI diagnosis were not well defined and studies in humans were lacking (12). In a 2018 study, various diagnostic criteria for rUTIs were compared across studies, highlighting the need for one clearly defined, uniform diagnostic criteria (13). With the increase in both antibiotic resistant infections and T2DM across the world, understanding the relationship between recurrent urinary tract infections and diabetes is crucial (1, 14). Given this context and several gaps in knowledge, our goal was to analyze current literature to understand where rUTI research in T2DM populations stands. We aimed to identify all English language articles on the topic of recurrent urinary tract infections in adult, type II diabetic women and compare research outcomes across studies on rUTI diagnostic criteria, rUTI incidence rates, characterization of rUTIs, risk factors for rUTIs, workup and diagnostic methods, UTI treatment durations, antibiotic prescription rates, antibiotic resistances, and the correlation of SGLT2 inhibitor use with rUTIs. We hypothesized that rUTI incidence rates would be higher in T2DM women than non-T2DM women.

## Methods

### Study design

We aimed to collect all studies with relevant information on the workup, characteristics, and treatment of rUTIs in women with T2DM including data on rUTI incidence rates, common bacterial strains, symptoms of infection, risk factors for rUTI, rUTI treatments, and post-treatment outcomes.

### Systematic review

A systematic review was performed in accordance with Cochrane and Preferred Reporting Items for Systematic Reviews and Meta-Analyses (PRISMA) standards (15). We reviewed articles published from inception to June 2022 in PubMed/MEDLINE, OVID Embase, and Cochrane Library. The references of relevant articles were hand searched by the reviewers to identify any additional articles. The study criteria outlined below were used in this review:

### Inclusion criteria

- Full text, English-language, prospective cohort, retrospective cohort, and randomized control trial studies of adult female patients.
- Studies focused on rUTI and T2DM; studies with an initial focus on UTI with relevant, clearly defined rUTI data, were included.

### Exclusion criteria

- Abstract-only texts, individual case reports, review articles, non-human studies. Review articles were not excluded until full-text examination and reference screening to ensure comprehensive article identification.
- Studies with strictly male or pediatric populations; due to the limited dataset, studies including men or pediatric patients were not excluded if the study included a significant proportion of women. Such studies were reported separately.
- Asymptomatic bacteriuria, pyelonephritis, and unspecified genitourinary infections.
- Type I diabetic populations only; due to the limited dataset, groups with both Type I and Type II diabetes were included and independently analyzed.

The search was conducted using the keywords [recur*] AND [urinary tract infection*] AND [diabetes] including MeSH terms. Alternative spellings, names, and abbreviations, such as “rUTI”, “chronic”, cystitis”, “T2DM” and “adult-onset diabetes” were thoroughly searched in all combinations. Additional keywords for diabetes medications such as “metformin,” “Ozempic,” and “sodium-glucose co-transporter 2 inhibitor” were used as alternative terms to find all possible additional diabetic populations. Keywords appeared at least once in the title, abstract, keywords, or full text. Findings were compared between reviewers and differences were reconciled after careful examination and discussion.

## Results

Through a multi-database and cross-reference search, 3342 records were identified. The titles of the articles were reviewed by two independent reviewers and were excluded if they did not meet study criteria or were duplicates. This step yielded 597 abstracts which were then further reviewed. 153 full-text studies were assessed for initial eligibility and were excluded based on format and topic exclusions. A total of 15 articles met all eligibility criteria.

**Figure 1:**
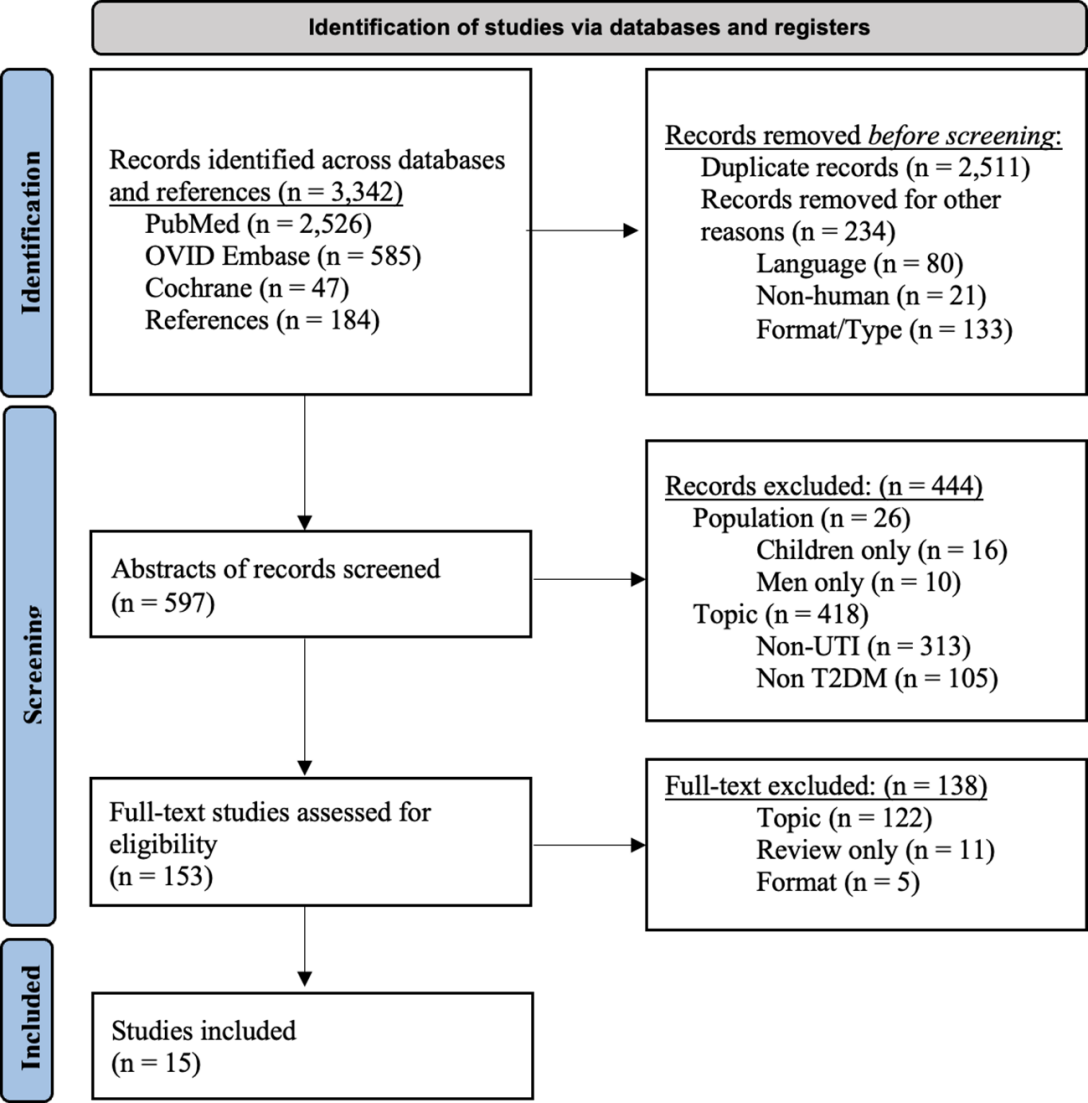
PRISMA 2020 flow diagram for new systematic reviews which included searches of databases and registers only. Adapted from: Page MJ, McKenzie JE, Bossuyt PM, Boutron I, Hoffmann TC, Mulrow CD, et al. The PRISMA 2020 statement: an updated guideline for reporting systematic reviews. BMJ 2021;372:n71. doi: 10.1136/bmj.n71. UTI, urinary tract infection; rUTI, recurrent urinary tract infection; T2DM, type II diabetes mellitus.

Fifteen studies were included in the final review (**Figure 1**). This included 4 prospective studies, 10 retrospective studies, and 1 study that was both prospective and retrospective. The countries of origin were the United States (n = 3), Netherlands (n = 2), Taiwan (n = 2), India (n = 2), Germany (n = 1), Greece (n = 1), Spain (n = 1), Israel (n = 1), Japan (n = 1), and Italy (n = 1).

### RUTI diagnosis criteria and incidence rates

The definitions and diagnostic criteria of recurrent UTI are included for each study (**Table 1**). Ten studies defined rUTI with a combination of symptomatology, urine culture, and prescription patterns and five studies used only one criterion. Nine studies set time frames in which multiple UTI diagnoses had to be made to consider the infection “recurrent”, most commonly 1 year.

**Table 1:** Overview of current RUTI and diabetes studies including women.

Year, Author & Study Type	RUTI definition	Objective	Patient number (% female)	Population	Ages	Groups	Selection process	Recurrence Rates	Strain	Symptoms	Risk factors	Workup, Treatment & Outcomes
2022 (R) Lin YH (16)	GUTI after first GUTI and stopping SGLT2i for min. 14D; GUTI occurrence after rechallenging SGLT2i for 7+D	Risk factors of SGLT2i related GUTI	103304 (43)	Adults with T2DM taking one SGLT2 inhibitor	18	T2	DM to UTI	28.2%.	NA	NA	DM with eGRF < 45 ml/min/1.73 m, CHD, and mood disorder (after rechallenging SGLT2i)	After first UTI, 63.5% of patients continued SGLT2i treatment, 9.82% discontinued SGLT2 inhibitors, 26.68% received SGLT2i rechallenge. 23/438 UTI patients needed IV antibiotics.
2018 (R) Lorenzo (17)	Undefined	SGLT2i discontinuation due to rUTIs in T2DM	691 (82)	Adults with SGL2 prescription	18+	T2	DM to UTI	NA	NA	NA	NA	2.5% of patients interrupted SGLT2 treatment due to rUTIs. Median treatment duration 8.8M. 58.8% received dapagliflozin, 29.4% empagliflozin, 11.8% canagliflozin.
2015 (R) Wilke (18)	Time to a second UTI in 1Y	Risk factors associated with UTI incidence/recurrence in T2DM	456586 (56)	Adult with T2DM	20+	T2	DM to UTI	NA	79.8% *E. coli/enterobacterial*, 24.0% by *Streptococcus group D*, 12.9% by *Staph. aureus*, 8.7% by *Pseudomonas*/non-fermenters, and 6.1% by other *Staphylococcus*.	NA	T2DM women 80+ years faced a 4-fold higher UTI/rUTI event risk than a 60-year-old man. Insulin treatment was associated with a higher UTI risk but not rUTI risk.	NA
2011 (R) Lin TL (19)	2+ episodes in 6M	Diversity of urodynamic findings and temporal effects on bladder dysfunction in DM; predisposing factors that attenuate the storage/voiding function of women with DM	181 (100)	T2DM women with LUTD	18+ (24-87)	T2	DM to UTI	DM 37%	NA	NA	NA	NA
2019 (P/R) Moustakas (20)	2 UC+ and symptomatic episodes within 6M or 3+ episodes in 1Y	Epidemiology of rUTIs and antimicrobial resistance patterns	100 (83)	Adults with urogenital symptoms	18+	DM, N	UTI to DM	DM 3-fold-risk for 3+ UTIs; 6.82% with ≤2 UTIs had DM, 28.57% with 3+ UTIs had DM	*E. coli* 96.2% (95.5% DM vs 85.7% non-DM)	3+ UTIs had higher rates of abdominal pain, urinary urgency, and less genital symptoms	30+ years of age, abnormal blood glucose levels, history of DM, more than one sexual partner in last 3 months, urinary urgency	Resistance to colistin and imipenem associated with 2+ UTIs. Most common anti-microbial was levofloxacin. Except for levofloxacin, no other resistance in antibiotics was significantly associated with the number of UTIs.
2019 (R) Vinod (21)	UC+ criteria for UTI, recurrence undefined	Epidemiology of rUTIs and antimicrobial resistance patterns	305 (56)	Culture positive adults +/- DM	18+	T1, T2, N	UTI to DM	DM 14.4%, non-DM 10.5% *	NA	NA	NA	Antibiotic resistance patterns similar in patients with and without DM.
2017 (R) Grigoryan (22)	UTI plus a new Rx for an antibiotic, 6-29D (early), or 30D - 1Y(late)	UTI management in women with and without DM, effect of treatment duration on early and late recurrence	1845 (100)	Adult women with UTI diagnosis	18+	DM, N	UTI to DM	NA	NA	NA	DM, higher risk of early recurrent with longer treatment durations for women +/- DM	Longer treatment associated with a 2-fold increase risk of rUTI and not protective against late recurrence (independent of DM). Women with DM had a higher risk of late recurrence, but longer treatment did not alter this risk.
2015 (R) Nseir (23)	Symptomatic UTI after resolution of previous UTI, or 3+ symptomatic episodes in 1Y	Association between obesity and recurrent UTIs (RUTIs) among premenopausal women	244 (100)	Premenop. women with RUTI	20-55	DM, N	UTI to DM	Premenop. women with UTI 23.4%	NA	NA	Obesity was associated with RUTIs in premenop. women independent of contraceptive use, age, sexual intercourse, DM and metabolic syndrome	NA
2014 (R) Fu (8)	Multiple UTI, cystitis, pyelonephritis claims (min 3+M apart)	Risk of UTI in subjects with newly diagnosed T2DM	89790 (49)	Adults with newly diagnosed T2DM, paired controls	18+	T2, N	DM to UTI	T2DM 1.6%, non-T2DM 0.6%	NA	NA	NA	NA
2014 (P) Aswani (24)	UC+ for UTI, recurrence undefined	Clinical and microbiological features of UTI in DM and non-DM; influence of DM on the uropathogens and antibiotic sensitivity pattern in UTI	305 (56)	Culture positive adults +/- DM	18+	DM, N	UTI to DM	NA	ESBL *E. coli* significantly higher in DM compared to non-DM. Frequency of pathogens: *E. coli, Klebsiella, Enterococcus* in both DM and non-DM	No significant difference in clinical symptoms between DM and non-DM subjects.	HbA1c > 8.0% in patients with DM	Antimicrobial resistance pattern similar in both DM and non-DM groups with max. sensitivity to meropenem and worst to ampicillin. Aminoglycosides showed better sensitivity profile in both DM and non-DM.*
2013 (R) Yoon (25)	3+ symptomatic episodes over 1Y (10^3 CFU/ml uropathogen midstream)	Risk factors of recurrent cystitis patients following acute cystitis	344 (100)	Women treated for cystitis	18-65	DM, N	UTI to DM	NA	Pathogen freq. in order: *E. coli*, *Staphylococcus Saprophyticus*, *Enterococcus*, *Klebsiella*. No statistically significant differences between groups.	NA	DM affected progression to recurrent cystitis from acute cystitis	NA
2013 (R) Grandy (26)	3+ episodes in 1Y	Prevalence, recurrence, and predisposing factors of self-reported UTI	10410 (65)	Adults +/- T2DM	18+	T2, N	DM to UTI	T2DM (min 1 UTI) 25%	NA	NA	Overactive bladder/incontinence, kidney problems, narrow or blocked arteries	NA
2010 (R) Gorter (27)	Relapses (represcribed antibiotics 4D- 6W after first Rx); reinfections (new Rx after 6W)	Prevalence, recurrence, and predisposing factors of self-reported UTI	7063 (100)	Women +/- DM	30+	T1, T2, N	DM to UTI	DM 7.1/15.9%, non-DM 2.0/4.1%	NA	NA	DM, duration of DM, treatment (insulin), retinopathy	Pattern of antibiotic prescription significantly different between first and recurrent episodes of UTI. Diabetes did not influence the antibiotic prescription pattern.
2008 (R) Schneeberger (28)	Re-Rx for medications (5-30D after first Rx) or hospitalization admission with diagnosis of UTI (relapses or reinfections)	Treatment strategies with respect to rUTI rates in women with and without DM	210624 (100)	Women +/- DM	12+	DM, N	DM to UTI	Premenop DM 16.1%, non-DM 12.2%; Postmenop. DM 19.1%, non-M 16.4%	NA	NA	NA	Prem./postm. women with DM receive longer and more potent antimicrobial treatment for UTIs compared to non-DM. Despite more aggressive treatment, those with DM have more recurrences of their UTIs.
2000 (P) Bonadio (29)	UC+ diagnosis for UTI, recurrence undefined	Epidemiological, microbiological, and clinical features of UTI in patients with and without DM	490 (70)	Individuals +/-DM	NA (1-89)	DM, N	UTI to DM	DM 52.8%, non-DM 42.9%*	DM vs non-DM: *E. coli* (56.1 vs. 56.8%), *Proteus* sp. (7.9% vs. 7.2%), *Pseudomonas* sp. (6.7 vs. 8.2%), *Enterococcus* sp. (6.7 vs. 7.2%).	NA	NA	Hospital-acquired strains more resistant to antibiotics than community-acquired.

*not statistically significant.

(P), prospective study; (R), retrospective study; Premenop., premenopausal; Postmenop., Postmenopausal; D/M/Y, days/months/years; SGLT2i, sodium-glucose cotransporter 2 inhibitors; GUTI, genitourinary tract infection; Rx, medical prescription; LUTD, lower urinary tract dysfunction; UC+, urine culture positive. NA, not applicable.

Twelve studies reported rUTI incidence rates. Grandy et al. reported both UTI and rUTI rates for their T2DM group, meanwhile Fu et al. studied general rUTI diagnosis rates in newly diagnosed T2DM patients. Other studies looked at recurrence rates in diabetic vs non-diabetic populations after a UTI diagnosis. Schneeberger et al. and Gorter et al. both found that women with DM had higher recurrence rates than their non-DM counterparts, however, neither of these studies distinguished between Type I and Type II diabetic patients. In contrast, two studies did not find significant differences in diabetic vs non-diabetic rUTI rates; once again, these studies did not differentiate Type 1 Diabetes Mellitus (T1DM) from T2DM.

### Characterization of RUTI

Of the five studies that reported specific strains, all 5 found *Escherichia coli (E. coli)* to be the most frequent causative agent for UTI (56.1% - 96.2%) both in diabetics and non-diabetics. None of the studies reported a significant difference between the two groups. Two studies reported pathogen rates in their studies specific to rUTI, both of which found *E. coli* to be the most frequently rUTI pathogen. Aswani et al. reported a higher prevalence of extended-spectrum beta-lactamase (ESBL) *E. coli* in diabetics vs. non-diabetics; in contrast, Yoon et al. found no significant differences in pathogen distribution between groups.

### Risk factors for RUTI

Diabetes has been shown to be a risk factor for UTI across many former studies (11). Of the 15 studies included in this review, five studies sought out to determine if diabetes was a risk factor for developing recurrent UTI, specifically. Yoon et al. found that having diabetes was significantly associated with the progression of acute to recurrent cystitis while Moustakas et al. found that DM was a risk factor for rUTI. Similarly, Grigoryan et al. found that the presence of DM was a determinant of late recurrence of UTI. For studies looking at risk factors for rUTI in diabetic populations, results varied greatly. Gorter et al. reported several risk factors for rUTI in women, including insulin treatment. Contrary to this finding, Wilke et al. reported that insulin treatment was not associated with rUTI risk.

### Workup, diagnosis, and treatment durations

Eight studies discussed the diagnosis, treatment, or outcomes of rUTIs in diabetes. Grigoryan et al. reported that women with diabetes and acute cystitis were less likely to receive workup for new cystitis events but were more likely to receive longer durations of antibiotics. They also found that treating UTI episodes for longer did not correlate with lower rates of recurrence. Similarly, Schneeberger et al. reported that women with DM received longer and more potent antimicrobial treatment for UTIs but had higher recurrence rates than non-DM women.

### Antibiotic prescription rates

Gorter et al. found that both women with and without DM received significantly different antibiotic prescriptions between their first episode of UTI and their recurrent episodes, but that diabetes did not influence antibiotic prescription patterns. Moustakas and Grigoryan et al. both reported that fluoroquinolones were the most prescribed antibiotic class; the latter also found that there was no clinically meaningful difference in prescription patterns between DM status groups. Similarly, Schneeberger et al. found no statistically significant difference in fluoroquinolone prescription between groups but reported that postmenopausal patients with DM were more likely to receive norfloxacin with longer treatment duration.

### Antibiotic resistance

Antibiotic resistance patterns were discussed in three studies. Aswani et al. reported similar patterns in both DM and non-DM populations; similarly, Bonadio et al. reported slight differences that did not reach statistical significance. Neither of these studies specified the impact of antibiotic resistance on rUTIs. Moustakas et al. looked at how resistance to antimicrobials was associated with multiple UTIs. They found that resistance to colistin and imipenem was associated with a history of >2 UTI episodes but observed only in a few patients.

### SGLT2 inhibitors

Two studies focused on the effects of sodium-glucose co-transporter 2 inhibitors (SGLT2i) on UTIs and their recurrences. The study by Lin YH et al. investigated the risk factors related to genitourinary tract infections with SGLT2i use. The authors found a 28.2% recurrence rate. The other study, Lorenzo et al. found that 10 out of 691 patients interrupted their SGLT2i use due to rUTIs.

## Risk of bias assessment

The Joanna Briggs Institute (JBI) critical appraisal checklist was used to analyze the risk of bias for the thirteen cohort studies and two cross-sectional analyses in this review. The results are summarized in (**Table 2**). The studies included in this review were not focused on acute treatment of a single UTI infection, and as a result, certain criteria for the risk of bias assessment categories were not applicable. The articles otherwise scored highly for similarities in the groups, exposure measurement, and statistical analysis. Some of the studies did not identify and/or address confounding variables, however, these studies were still high enough quality for inclusion.

**Table 2:** Risk of bias assessment for cohort and analytical cross-sectional studies.

Reference	Criteria for inclusion clearly defined	Subjects and setting described in detail	Exposure measured in a valid way	Objective measurement of condition	Confounding factors identified	Strategies to deal with confounding factors stated	Outcomes measured in a valid way	Appropriate statistical analysis used	Overall appraisal
Lin, YH (16)	Yes	Yes	Yes	No*	No	No	Yes	Yes	Include
Lorenzo (17)	Yes	No	Yes	Yes	No	No	Yes	Yes	Include
Wilke (18)	Yes	Yes	Yes	Yes	No	No	Yes	Yes	Include
Lin, TL (19)	Yes	Yes	Yes	Yes	No	No	Yes	Yes	Include
Moustakas (20)	Yes	Yes	Yes	Yes	Yes**	Yes	Yes	Yes	Include
Vinod (21)	Yes	No	Yes	Yes	No	No	Yes	Yes	Include
Grigoryan (22)	Yes	Yes	Yes	Yes	Yes**	Yes	Yes	Yes	Include
Nseir (23)	Yes	Yes	Yes	Yes	Yes	No	Yes	Yes	Include
Fu (8)	Yes	Yes	Yes	No*	No	No	Yes	Yes	Include
Aswani (24)	Yes	No	Yes	Yes	No	No	Yes	Yes	Include
Yoon (25)	Yes	Yes	Yes	No*	No	No	Yes	Yes	Include
Grandy (26)	Yes	Yes	Yes	Yes	Yes	Yes	Yes	Yes	Include
Gorter (27)	Yes	Yes	Yes	No*	Yes	Yes	Yes	Yes	Include
Schneeberger (28)	Yes	Yes	Yes	No*	Yes	Yes	Yes	Yes	Include
Bonadio (29)	Yes	Yes	Yes	Yes	No	No	Yes	Yes	Include

*UTI episodes not confirmed through culture.

** Confounders partially identified.

## Discussion

This systematic review of existing literature on rUTIs in women with diabetes was done according to PRISMA guidelines. In addition to a relative dearth of publications, we observed that only two studies reported exclusively on rUTIs in T2DM women. The others included UTI data as well as information on diabetic men. RUTI definitions were heterogeneous and most did not comply with the more recently adopted criteria of two symptomatic UTIs in six months of three in a year (13). In the end, fifteen articles were identified as relevant to this topic, covering various subtopics including rUTI incidence rates, characteristics, symptoms, risk factors, treatments, and outcomes.

RUTI incidence rates in diabetic populations were the most reported findings across the studies. Overall, four studies reported non-comparative recurrence rates ranging from 23.4% to 37% in different diabetic populations. Six studies compared rUTI rates between populations, most commonly diabetics vs. non-diabetics. Four of these yielded statistically significant differences between groups, although not all studies fully addressed possible confounding variables. Two studies found differences in rUTI rates between diabetics and non-diabetics that were not statistically significant. These differences between studies are possibly the result of variable diabetes groups (T2DM vs all diabetics), small study populations, short study durations, and a lack of rUTI focus.

Another aim of this review was to assess the risk factors for rUTI. Four of five studies found that diabetes was a determining risk factor for rUTI, but no specific conclusion can be drawn regarding T2DM as a risk factor, specifically. Additional risk factors for rUTI in diabetic patients mentioned across the studies included retinopathy, overactive bladder, incontinence, kidney problems, narrow/blocked arteries, insulin treatment, age, and duration of diabetes. However, many of these findings were inconsistent across studies. This is again likely the result of the differences in study populations, methodology, and rUTI definitions.

Antibiotic usage and resistance were the focus of treatment data across studies. Due to the varying geographical locations of the studies included in this review, guidelines for antibiotic prescriptions varied greatly, limiting our ability to compare antibiotic prescription findings. Despite these differences, it was reported that longer durations of antibiotic treatment in diabetic patients does not correlate with less UTI recurrences. Otherwise, no significant findings were reported for antibiotic resistance patterns between diabetic and non-diabetic groups.

*E. coli* was found to be the most frequent causative agent of both UTI and rUTI across studies, both for diabetic and non-diabetic groups. Only one study found a significant difference between the causative agent of UTI/rUTI in diabetic and non-diabetic groups; ESBL was determined to be higher in diabetics. For symptoms of rUTI specifically, only one study reported relevant findings. Moustakas et al. found that urinary urgency, abdominal pain, and the absence of genital symptoms were correlated with having ≥3 UTIs in a year.

## Areas of gaps of knowledge

As underscored by this review, several gaps in knowledge in the field of rUTI research in diabetics were identified. To our knowledge, this review is the first formal systematic review of the limited literature available on the topic of recurrent urinary tract infections in type II diabetics, with a focus on female populations. This project was initiated to better understand the gaps in knowledge in this growing field and aging population. Although there has been a recent suggestion for a standardized definition of rUTI, many of the studies in this review used different diagnostic criteria. In addition, there were so few rUTI studies in T2DM women specifically, that studies with unspecified types of diabetes (T1DM and T2DM not separated) and studies that included some men had to be included. Due to these large differences in study populations, methodologies, and aims, performing a valuable statistical comparison between studies, such as a meta-analysis, was not possible. UTI recurrence and incidence rates were difficult to compare. Additionally, treatment options varied greatly between countries because of guidelines as well as high rates of antibiotic-resistant organisms and antibiotic allergies (6, 13, 18, 20–22, 28). This resulted in an inability to compare treatment results across studies. Lastly, patients with diabetes often had several comorbidities that were difficult to control for, and multiple studies did not identify confounding variables.

## Conclusions

This systematic review summarizes the literature on recurrent urinary tract infections in diabetic women. Fifteen studies from 10 countries met study criteria, providing a heterogenous population. The included articles covered subtopics from recurrent UTI rates, risk factors, symptoms, characteristics, treatments, and disease outcomes. Several studies focused on UTIs and diabetes as their primary goal, and recurrent UTIs only as their secondary target. Hence, rUTI specific results included in this review were limited and not generalizable. However, multiple studies found diabetes to be a risk factor for rUTI, supporting our initial hypothesis that rUTI rates are higher in diabetics than non-diabetics. The findings of this review indicate an urgent need for more research, specifically well-structured prospective studies to determine how best to evaluate and manage recurrent urinary tract infections in diabetic patients.

## Data availability statement

The original contributions presented in the study are included in the article/supplementary material. Further inquiries can be directed to the corresponding author.

## Author contributions

SP: Data curation, Formal Analysis, Investigation, Methodology, Writing – original draft, Writing – review & editing. PZ: Conceptualization, Data curation, Formal Analysis, Investigation, Methodology, Project administration, Resources, Writing – original draft, Writing – review & editing.
